# Crystal structure of 3-[({2-[bis­(2-hy­droxy­benz­yl)amino]­eth­yl}(2-hy­droxy­benz­yl)amino)­meth­yl]-2-hydroxy-5-methyl­benzaldehyde

**DOI:** 10.1107/S1600536814024465

**Published:** 2014-11-21

**Authors:** Alexandra S. Fonseca, Adailton J. Bortoluzzi

**Affiliations:** a Depto. de Química – Universidade Federal de Santa Catarina, 88040-900 Florianópolis, Santa Catarina, Brazil

**Keywords:** crystal structure, non-symmetrical compound, tetra­substituted ethyl­enedi­amine, phenol-arm substituents

## Abstract

The non-symmetric title mol­ecule, C_32_H_34_N_2_O_5_, is based on a tetra­substituted ethyl­enedi­amine backbone. The mol­ecular structure consists of three hy­droxy­benzyl groups and one 2-hy­droxy-5-methyl­benzaldehyde group bonded to the N atoms of the di­amine unit. The ethyl­enedi­amine skeleton shows a regular extended conformation, while the spatial orientation of the phenol arms is governed by hydrogen bonds. In the 2-hy­droxy-5-methyl­benzaldehyde group, an intra­molecular *S*(6) O—H⋯O hydrogen bond is observed between the alcohol and aldehyde functions, and the neighbouring phenol arm participates in an intra­molecular *S*(6) O—H⋯N hydrogen bond. The third phenol group is involved in a bifurcated intra­molecular hydrogen bond with graph-set notation *S*(6) for O—H⋯N and O—H⋯O intra­molecular hydrogen bonds between neighbouring amine and phenol arms, respectively. Finally, the fourth phenol group acts as an acceptor in a bifurcated intra­molecular hydrogen bond and also acts as donor in an inter­molecular hydrogen bond, which connects inversion-related mol­ecules into dimers with *R*_4_^4^(8) ring motifs.

## Abstract 2

The mol­ecular structure of a non-symmetric structure based on a tetra­substituted ethyl­enedi­amine backbone consists of three hy­droxy­benzyl groups and one 2-hy­droxy-5-methyl­benzaldehyde group bonded to the N atoms of the di­amine unit. The ethyl­enedi­amine skeleton shows a regular extended conformation, while the phenol arms are randomly oriented but governed by hydrogen bonds.

## Chemical context   

The preparation of non-symmetric compounds has always been of inter­est in organic synthesis, as well as in coordination chemistry. Compounds containing tetra­substituted ethyl­ene­di­amine groups have attracted significant inter­est because of their coordination versatility towards metal ions, their easy preparation and their biological activity (Musa *et al.*, 2014 ▶). With respect to medical applications, high *in vitro* cytotoxic activity of free ethyl­enedi­amine-type compounds against different types of cancer cells, such as HL-60 leukemic and B16 human melanoma cells lines, has been reported (Dencic *et al.*, 2012 ▶; Lazić *et al.*, 2010 ▶). In addition, metal complexes containing substituted ethyl­enedi­amine have also found valuable applications in pharmacological research as potential anti­cancer agents (Ansari *et al.*, 2009 ▶), radiopharmaceuticals for tumor imaging (Boros *et al.*, 2011 ▶; Price *et al.*, 2012 ▶) and artificial nucleases (Raman *et al.*, 2011 ▶). In this paper, we report the synthesis and crystal structure of the non-symmetric mol­ecule 3-[({2-[bis­(2-hy­droxy­benz­yl)amino]­eth­yl}(2-hydroxy­benz­yl)amino)­meth­yl]-2-hy­droxy-5-methyl­benzaldehyde, (I), which is a potential hexa­dentate ligand with an N_2_O_4_-donor set which could stabilize complexes containing high-oxidation-state metal ions, such as Tc^III^, Ga^III^ and In^III^ ions, that are widely used in radiopharmaceuticals for diagnostic imaging and related research. 
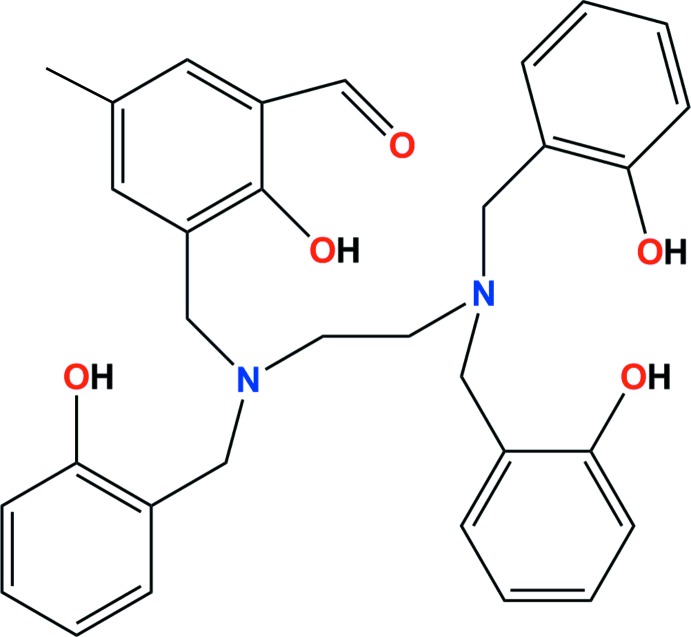


## Structural commentary   

**Figure 1:**
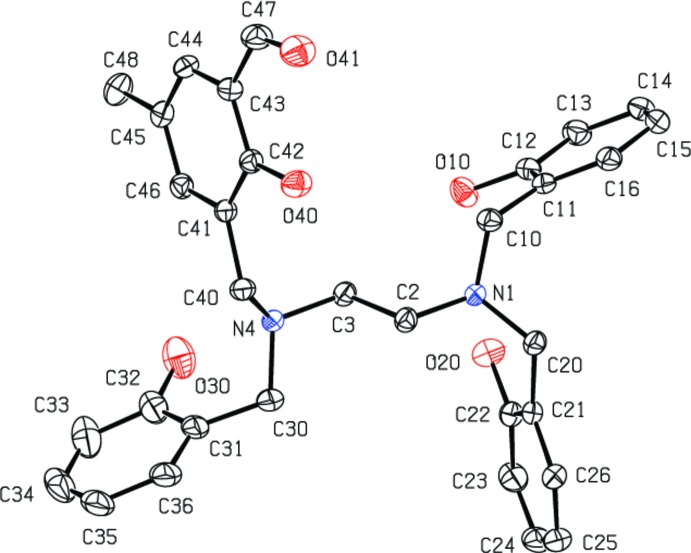
The mol­ecular structure of (I), with displacement ellipsoids drawn at the 40% probability level.

**Figure 2:**
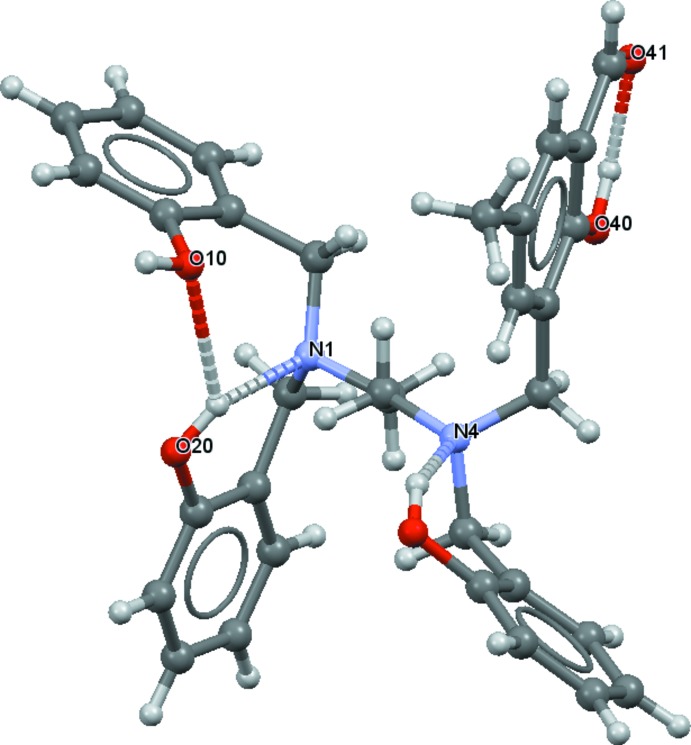
The intra­molecular hydrogen bonds (dashed lines) observed in (I).

Compound (I) is a non-symmetric mol­ecule based on a tetra­substituted ethyl­enedi­amine backbone (Fig. 1 ▶). The structure consists of three hy­droxy­benzyl groups and one 2-hy­droxy-5-methyl­benzaldehyde group bonded to nitro­gen atoms of the di­amine unit. The ethyl­enedi­amine skeleton shows a regular extended ‘zigzag’ conformation [with an N1—C2—C3—N4 torsion angle of 174.78 (13)°], while the pendant phenol arms are randomly oriented but governed by hydrogen bonds (Table 1 ▶). Three intra­molecular hydrogen bonds with an *S*(6) graph-set motif are observed in the mol­ecular structure of (I) (Fig. 2 ▶). One of these occurs between the neighbouring alcohol and aldehyde groups. In addition, intra­molecular O—H⋯N and O—H⋯O inter­actions, which include bifurcated hydrogen bonds, are observed, involving O—H functions as donors and the amine sites and one phenolic oxygen atom as acceptors. All bond lengths and angles found for (I) are in the expected range for organic compounds (Bruno *et al.*, 2004 ▶).

## Supra­molecular features   

**Figure 3:**
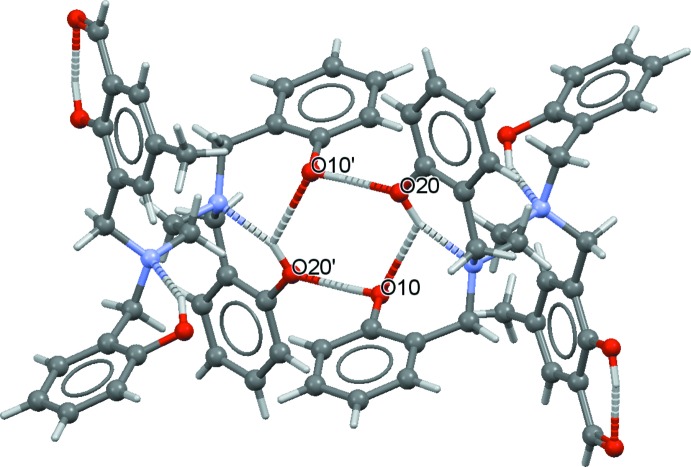
An inversion dimer of (I) formed by inter­molecular O—H⋯O hydrogen bonds (dashed lines). [Symmetry code: (′) −*x* + 1, −*y*, −*z*.]

**Figure 4:**
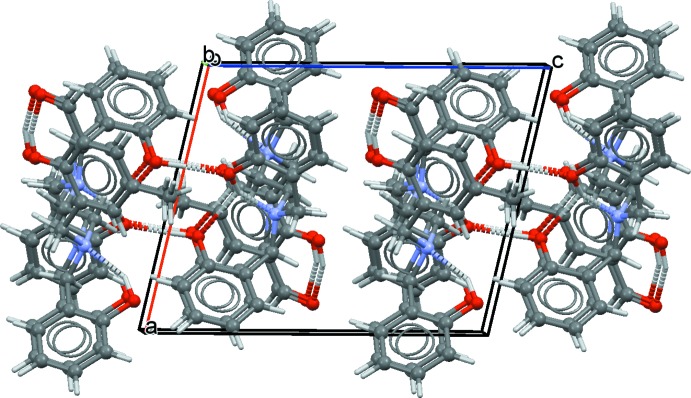
Partial packing of (I), showing dimers stacked along [010].

In the crystal of (I), inversion dimers with 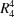
(8) ring motifs are formed by pairs of O—H⋯O hydrogen bonds (Fig. 3 ▶, Table 1 ▶). The approximate planes of the ring motifs of the dimers are arranged as stacks along [010] (Fig. 4 ▶). No π–π stacking inter­actions are observed.

## Database survey   

A search for similar structures in the current version of the Cambridge Structural Database (Version 5.35, November 2013; Groom & Allen, 2014 ▶) resulted in four entries but only three different structures: (i) HUNDIE (CCDC 727272) and HUNDOK (CCDC 727273) (Boyle *et al.*, 2009 ▶); (ii) USODUC (CCDC 809654) (Wang *et al.*, 2011*a* ▶) and (iii) USODUC01 (CCDC 809654) (Wang *et al.*, 2011*b* ▶). All of these structures are symmetric mol­ecules and the phenol groups have an additional one or two substituents in the *para* and *ortho* positions with respect to the O–H function. As observed in (I), the spatial orientations of the phenol arms are influenced by intra- and inter­molecular hydrogen bonding. There are no significant differences in the geometrical parameters; however, the crystal packing shows distinguishable three-dimensional arrangements due to differences in mol­ecular symmetry and inter­molecular inter­actions.

## Synthesis and crystallization   

The title compound was obtained from a nucleophilic substitution reaction between *N*,*N*,*N*′-tris­(2-hy­droxy­benz­yl)-1,2-di­amino­ethane (Schmitt *et al.*, 2002 ▶) and chloro­methyl-4-methyl-6-formyl­phenol. These precursors were prepared following the methodologies already described in the literature (Schmitt *et al.*, 2002 ▶; Thoer *et al.*, 1988 ▶). A solution of 2-chloro­methyl-4-methyl-6-formyl­phenol (1.19 g, 6.6 mmol) in tetra­hydro­furan (40 ml) was added slowly to a cooled solution of *N*,*N*,*N*′-tris­(2-hy­droxy­benz­yl)-1,2-di­amino­ethane (2.50 g, 6.6 mmol) in tetra­hydro­furan (40 ml) containing tri­ethyl­amine (0.96 ml, 6.6 mmol). The reaction was kept cooled during addition time, and the resulting solution stirred for 24 h. Yellow mixture oil/solid was obtained after evaporation of the solvent. A solution of this mixture in CH_2_Cl_2_ (50 ml) was washed with a saturated solution of NaHCO_3_ (3 × 50 ml) and filtered off in the presence of NaSO_4_. The solvent was removed, and a straw-yellow solid was obtained. This solid was refluxed in *n*-hexa­ne/CHCl_3_ (1:1, 100 ml). After cooling the solid was filtered off, washed with *n*-hexane (80 ml), dried and recrystallized from an ethyl acetate solution to afford 3-[({2-[bis­(2-hydroxy­benz­yl)amino]­eth­yl}(2-hy­droxy­benz­yl)amino)meth­yl]-2-hydroxy-5-methyl­benzaldehyde, (I).

The formation of (I) was indicated by the presence of the band at 1655 cm^−1^ in the IR spectrum, which is typical for stretching vibrations ν(C=O) of free aldehyde. In the ^1^H NMR spectrum, the signal at 9.81 p.p.m. related to one aldehyde proton is further evidence for product formation. Yield 90%, m.p. 444.8–445.4 K. IR (KBr, cm^−1^): ν(O—H) 3273, ν(C—H_ar_ and C—H_alif_) 3042–2718, ν(C=O)1655, ν(C=C) 1615–1457, δ(O—H) 1365, δ(C—O) 1252, δ(C—H_ar_) 757; ^1^H NMR (400 MHz, CDCl_3_) (δ, p.p.m.): 2.29 (*s*, 3H, CH_3_), 2.78 (*s*, 4H, CH_2-en_), 3.58 (*s*, 2H, CH_2_), 3.61–3.77 (*m*, 6 H, CH_2_), 6.69–6.87 (*m*, 6H, CH_ar_), 6.91 (*d*, 2H, CH_ar_), 6.99 (*d*, 2 H, CH_ar_), 7.07–7.19 (*m*, 2H, CH_ar_), 7.24 (*d*, 2H, CH_ar_), 9.81 (*s*, 1H, CH_ald_); ^13^C NMR (400 MHz, DMSO-*d*_6_, δ p.p.m.): 20.0, 48.6, 48.8, 53.5, 54.3, 115.2, 121.7, 122.7, 123.2, 124.5, 127.6, 128.4, 128.7, 129.8, 130.8, 136.7, 156.2, 156.5, 158.7, 191.6. Negative HPLC/ESI–MS (*m*/*z*): [*M*−H] calculated for C_32_H_35_N_2_O_5_^−^, 527.25; found, 527.19. Colourless blocks were grown by slow evaporation of the solvent from a saturated solution of (I) in ethyl acetate.

## Refinement details   

Crystal data, data collection and structure refinement details are summarized in Table 2 ▶. H atoms were placed in idealized positions with distances of 0.95 (CH_Ar_), 0.99 (CH_2_) or 0.98 Å (CH_3_) with *U*_iso_ = 1.2*U*_eq_(C) or 1.5*U*_eq_(C_meth­yl_). The hydrogen atoms of the alcohol groups were located from a Fourier difference map and treated with a riding-model approximation with *U*_iso_(H) = 1.5*U*_eq_(O).

## Supplementary Material

Crystal structure: contains datablock(s) general, I. DOI: 10.1107/S1600536814024465/lh5739sup1.cif

Structure factors: contains datablock(s) I. DOI: 10.1107/S1600536814024465/lh5739Isup2.hkl

Supporting information file. DOI: 10.1107/S1600536814024465/lh5739Isup3.mol

Supporting information file. DOI: 10.1107/S1600536814024465/lh5739Isup4.cml

CCDC reference: 1033129

Additional supporting information:  crystallographic information; 3D view; checkCIF report

## Figures and Tables

**Table 1:** Hydrogen-bond geometry (, )

*D*H*A*	*D*H	H*A*	*D* *A*	*D*H*A*
O10H10O20^i^	0.93	1.80	2.7230(16)	177
O20H20N1	0.94	1.75	2.5928(17)	148
O20H20O10	0.94	2.43	3.0362(19)	122
O30H30N4	0.93	1.94	2.784(2)	149
O40H40O41	0.93	1.76	2.6146(19)	151

Symmetry code: (i) 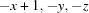.

**Table 2:** Experimental details

Crystal data	
Chemical formula	C_32_H_34_N_2_O_5_
*M*_r_	526.61
Crystal system, space group	Triclinic, *P* 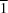
Temperature (K)	190
*a*, *b*, *c* ()	10.1635(5), 11.0440(6), 13.5439(7)
, , ()	113.549(2), 98.381(2), 99.451(3)
*V* (^3^)	1336.64(12)
*Z*	2
Radiation type	Mo *K*
(mm^1^)	0.09
Crystal size (mm)	0.15 0.08 0.04

Data collection	
Diffractometer	Bruker APEXII DUO
No. of measured, independent and observed [*I* > 2(*I*)] reflections	17177, 8122, 5175
*R*_int_	0.031
(sin /)_max_ (^1^)	0.715

Refinement	
*R*[*F* ^2^ > 2(*F* ^2^)], *wR*(*F* ^2^), *S*	0.057, 0.166, 1.02
No. of reflections	8122
No. of parameters	353
H-atom treatment	H-atom parameters constrained
_max_, _min_ (e ^3^)	0.42, 0.25

Computer programs: *APEX2* and *SAINT* (Bruker, 2009 ▶), *SHELXS97* and *SHELXL97* (Sheldrick, 2008 ▶), *PLATON* (Spek, 2009 ▶) and *Mercury* (Macrae *et al.*, 2008 ▶).

## supplementary crystallographic information

### Crystal data

**Table:**

C_32_H_34_N_2_O_5_	*Z* = 2
*M**_r_* = 526.61	*F*(000) = 560
Triclinic, *P*1	*D*_x_ = 1.308 Mg m^−^^3^
Hall symbol: -P 1	Mo *K*α radiation, λ = 0.71073 Å
*a* = 10.1635 (5) Å	Cell parameters from 4159 reflections
*b* = 11.0440 (6) Å	θ = 2.4–30.3°
*c* = 13.5439 (7) Å	µ = 0.09 mm^−^^1^
α = 113.549 (2)°	*T* = 190 K
β = 98.381 (2)°	Prismatic, colourless
γ = 99.451 (3)°	0.15 × 0.08 × 0.04 mm
*V* = 1336.64 (12) Å^3^	

### Data collection

**Table:**

Bruker APEXII DUO diffractometer	5175 reflections with *I* > 2σ(*I*)
Radiation source: fine-focus sealed tube	*R*_int_ = 0.031
Graphite monochromator	θ_max_ = 30.6°, θ_min_ = 1.7°
φ and ω scans	*h* = −13→14
17177 measured reflections	*k* = −15→15
8122 independent reflections	*l* = −9→19

### Refinement

**Table:**

Refinement on *F*^2^	Primary atom site location: structure-invariant direct methods
Least-squares matrix: full	Secondary atom site location: difference Fourier map
*R*[*F*^2^ > 2σ(*F*^2^)] = 0.057	Hydrogen site location: inferred from neighbouring sites
*wR*(*F*^2^) = 0.166	H-atom parameters constrained
*S* = 1.02	*w* = 1/[σ^2^(*F*_o_^2^) + (0.0806*P*)^2^ + 0.2575*P*] where *P* = (*F*_o_^2^ + 2*F*_c_^2^)/3
8122 reflections	(Δ/σ)_max_ < 0.001
353 parameters	Δρ_max_ = 0.42 e Å^−^^3^
0 restraints	Δρ_min_ = −0.25 e Å^−^^3^

### Special details

**Table:**

Refinement. Refinement of *F*^2^ against ALL reflections. The weighted *R*-factor *wR* and goodness of fit *S* are based on *F*^2^, conventional *R*-factors *R* are based on *F*, with *F* set to zero for negative *F*^2^. The threshold expression of *F*^2^ > 2σ(*F*^2^) is used only for calculating *R*-factors(gt) *etc*. and is not relevant to the choice of reflections for refinement. *R*-factors based on *F*^2^ are statistically about twice as large as those based on *F*, and *R*- factors based on ALL data will be even larger.

### Fractional atomic coordinates and isotropic or equivalent isotropic displacement parameters (Å^2^)

**Table:**

	*x*	*y*	*z*	*U*_iso_*/*U*_eq_	
N1	0.55546 (13)	0.19576 (13)	0.30195 (10)	0.0231 (3)	
C2	0.46867 (17)	0.29324 (17)	0.32893 (12)	0.0268 (3)	
H2A	0.5193	0.3775	0.3954	0.032*	
H2B	0.3853	0.2536	0.3460	0.032*	
C3	0.42744 (17)	0.32788 (16)	0.23213 (12)	0.0258 (3)	
H3A	0.5117	0.3735	0.2200	0.031*	
H3B	0.3865	0.2416	0.1646	0.031*	
N4	0.33030 (13)	0.41522 (13)	0.24463 (11)	0.0258 (3)	
C10	0.69979 (16)	0.26500 (17)	0.31867 (13)	0.0280 (3)	
H10A	0.7441	0.3071	0.3987	0.034*	
H10B	0.7017	0.3390	0.2948	0.034*	
C11	0.78117 (16)	0.17145 (16)	0.25636 (13)	0.0263 (3)	
C12	0.74930 (16)	0.11211 (18)	0.14067 (13)	0.0288 (3)	
C13	0.82640 (18)	0.02915 (19)	0.08148 (15)	0.0350 (4)	
H13	0.8023	−0.0122	0.0028	0.042*	
C14	0.93856 (18)	0.0067 (2)	0.13728 (16)	0.0368 (4)	
H14	0.9915	−0.0497	0.0967	0.044*	
C15	0.97344 (18)	0.06622 (19)	0.25181 (16)	0.0350 (4)	
H15	1.0507	0.0515	0.2901	0.042*	
C16	0.89474 (17)	0.14765 (18)	0.31045 (14)	0.0312 (4)	
H16	0.9188	0.1880	0.3891	0.037*	
C20	0.54425 (17)	0.11170 (17)	0.36403 (13)	0.0268 (3)	
H20A	0.5551	0.1718	0.4434	0.032*	
H20B	0.6192	0.0640	0.3576	0.032*	
C21	0.40800 (16)	0.00815 (16)	0.32108 (13)	0.0259 (3)	
C22	0.34574 (17)	−0.05756 (17)	0.20764 (14)	0.0296 (3)	
C23	0.22507 (19)	−0.15869 (19)	0.16750 (17)	0.0394 (4)	
H23	0.1832	−0.2018	0.0905	0.047*	
C24	0.1654 (2)	−0.1970 (2)	0.23937 (19)	0.0457 (5)	
H24	0.0838	−0.2678	0.2113	0.055*	
C25	0.2237 (2)	−0.1331 (2)	0.35144 (19)	0.0439 (5)	
H25	0.1822	−0.1592	0.4007	0.053*	
C26	0.34370 (18)	−0.03025 (19)	0.39182 (16)	0.0338 (4)	
H26	0.3827	0.0149	0.4693	0.041*	
C30	0.20423 (18)	0.36075 (19)	0.27170 (17)	0.0364 (4)	
H30A	0.2235	0.3827	0.3516	0.044*	
H30B	0.1767	0.2604	0.2293	0.044*	
C31	0.08900 (18)	0.41958 (19)	0.24471 (18)	0.0404 (4)	
C32	0.0497 (2)	0.4116 (2)	0.13898 (18)	0.0453 (5)	
C33	−0.0650 (2)	0.4543 (2)	0.1098 (2)	0.0613 (7)	
H33	−0.0926	0.4460	0.0368	0.074*	
C34	−0.1378 (2)	0.5087 (2)	0.1875 (3)	0.0637 (7)	
H34	−0.2174	0.5359	0.1672	0.076*	
C35	−0.0980 (2)	0.5243 (2)	0.2931 (3)	0.0603 (7)	
H35	−0.1472	0.5660	0.3467	0.072*	
C36	0.0148 (2)	0.4792 (2)	0.3227 (2)	0.0499 (5)	
H36	0.0416	0.4889	0.3962	0.060*	
C40	0.39032 (17)	0.55858 (16)	0.32520 (14)	0.0286 (3)	
H40A	0.4334	0.5613	0.3967	0.034*	
H40B	0.3159	0.6066	0.3376	0.034*	
C41	0.49612 (16)	0.63311 (16)	0.28855 (13)	0.0247 (3)	
C42	0.62638 (16)	0.70468 (16)	0.35788 (12)	0.0260 (3)	
C43	0.71949 (16)	0.78163 (17)	0.32493 (13)	0.0284 (3)	
C44	0.68364 (17)	0.78221 (17)	0.22159 (13)	0.0293 (3)	
H44	0.7473	0.8341	0.1998	0.035*	
C45	0.55741 (18)	0.70888 (17)	0.15050 (13)	0.0290 (3)	
C46	0.46489 (17)	0.63763 (17)	0.18737 (13)	0.0277 (3)	
H46	0.3762	0.5900	0.1406	0.033*	
C47	0.85585 (19)	0.8554 (2)	0.39565 (15)	0.0385 (4)	
H47	0.9159	0.9059	0.3704	0.046*	
C48	0.5214 (2)	0.7027 (2)	0.03654 (14)	0.0406 (4)	
H48A	0.5719	0.7864	0.0368	0.061*	
H48B	0.4226	0.6935	0.0152	0.061*	
H48C	0.5462	0.6241	−0.0166	0.061*	
O10	0.64008 (13)	0.13999 (14)	0.09024 (10)	0.0368 (3)	
H10	0.6275	0.1024	0.0139	0.044*	
O20	0.40399 (13)	−0.02278 (13)	0.13465 (9)	0.0364 (3)	
H20	0.4684	0.0616	0.1734	0.044*	
O30	0.12438 (17)	0.36127 (17)	0.06209 (13)	0.0586 (4)	
H30	0.2124	0.3804	0.1050	0.070*	
O40	0.66072 (13)	0.69757 (14)	0.45562 (10)	0.0380 (3)	
H40	0.7520	0.7475	0.4859	0.046*	
O41	0.89821 (14)	0.85675 (16)	0.48535 (11)	0.0491 (4)	

### Atomic displacement parameters (Å^2^)

**Table:**

	*U*^11^	*U*^22^	*U*^33^	*U*^12^	*U*^13^	*U*^23^
N1	0.0239 (6)	0.0241 (6)	0.0259 (6)	0.0070 (5)	0.0077 (5)	0.0144 (5)
C2	0.0319 (8)	0.0270 (8)	0.0272 (7)	0.0117 (7)	0.0117 (6)	0.0142 (6)
C3	0.0303 (8)	0.0247 (8)	0.0257 (7)	0.0085 (6)	0.0090 (6)	0.0128 (6)
N4	0.0230 (6)	0.0211 (6)	0.0359 (7)	0.0052 (5)	0.0086 (5)	0.0144 (5)
C10	0.0237 (8)	0.0273 (8)	0.0306 (8)	0.0022 (6)	0.0043 (6)	0.0125 (6)
C11	0.0215 (7)	0.0259 (8)	0.0335 (8)	0.0023 (6)	0.0066 (6)	0.0162 (7)
C12	0.0231 (8)	0.0338 (9)	0.0340 (8)	0.0060 (7)	0.0087 (6)	0.0189 (7)
C13	0.0317 (9)	0.0410 (10)	0.0359 (9)	0.0095 (8)	0.0128 (7)	0.0182 (8)
C14	0.0283 (9)	0.0389 (10)	0.0515 (10)	0.0116 (8)	0.0183 (8)	0.0235 (9)
C15	0.0233 (8)	0.0389 (10)	0.0512 (10)	0.0082 (7)	0.0082 (7)	0.0278 (9)
C16	0.0243 (8)	0.0341 (9)	0.0363 (8)	0.0027 (7)	0.0034 (6)	0.0193 (7)
C20	0.0279 (8)	0.0285 (8)	0.0280 (7)	0.0070 (6)	0.0057 (6)	0.0165 (6)
C21	0.0248 (8)	0.0242 (8)	0.0342 (8)	0.0090 (6)	0.0088 (6)	0.0165 (6)
C22	0.0294 (8)	0.0240 (8)	0.0371 (8)	0.0085 (7)	0.0097 (7)	0.0136 (7)
C23	0.0314 (9)	0.0274 (9)	0.0484 (10)	0.0037 (7)	0.0034 (8)	0.0088 (8)
C24	0.0287 (9)	0.0303 (10)	0.0757 (14)	0.0027 (8)	0.0142 (9)	0.0218 (10)
C25	0.0371 (10)	0.0409 (11)	0.0705 (14)	0.0126 (9)	0.0256 (10)	0.0352 (10)
C26	0.0336 (9)	0.0351 (9)	0.0443 (9)	0.0121 (7)	0.0149 (7)	0.0255 (8)
C30	0.0277 (9)	0.0319 (9)	0.0552 (11)	0.0045 (7)	0.0136 (8)	0.0242 (8)
C31	0.0225 (8)	0.0279 (9)	0.0699 (13)	0.0010 (7)	0.0098 (8)	0.0226 (9)
C32	0.0336 (10)	0.0280 (10)	0.0612 (12)	0.0057 (8)	0.0007 (9)	0.0106 (9)
C33	0.0442 (13)	0.0331 (11)	0.0821 (17)	0.0056 (10)	−0.0162 (11)	0.0127 (11)
C34	0.0277 (10)	0.0339 (12)	0.113 (2)	0.0043 (9)	0.0014 (12)	0.0225 (13)
C35	0.0338 (11)	0.0353 (11)	0.116 (2)	0.0094 (9)	0.0324 (13)	0.0314 (13)
C36	0.0362 (11)	0.0367 (11)	0.0832 (15)	0.0069 (9)	0.0258 (10)	0.0292 (11)
C40	0.0279 (8)	0.0253 (8)	0.0348 (8)	0.0062 (6)	0.0134 (7)	0.0135 (7)
C41	0.0255 (7)	0.0207 (7)	0.0305 (7)	0.0079 (6)	0.0109 (6)	0.0114 (6)
C42	0.0273 (8)	0.0266 (8)	0.0261 (7)	0.0077 (6)	0.0079 (6)	0.0125 (6)
C43	0.0243 (8)	0.0284 (8)	0.0317 (8)	0.0038 (6)	0.0058 (6)	0.0134 (7)
C44	0.0302 (8)	0.0262 (8)	0.0360 (8)	0.0048 (7)	0.0112 (7)	0.0175 (7)
C45	0.0336 (9)	0.0260 (8)	0.0300 (8)	0.0092 (7)	0.0077 (6)	0.0140 (6)
C46	0.0253 (8)	0.0251 (8)	0.0311 (8)	0.0057 (6)	0.0032 (6)	0.0119 (6)
C47	0.0283 (9)	0.0394 (10)	0.0428 (10)	−0.0015 (8)	0.0034 (7)	0.0184 (8)
C48	0.0518 (12)	0.0422 (11)	0.0324 (9)	0.0116 (9)	0.0078 (8)	0.0215 (8)
O10	0.0338 (7)	0.0523 (8)	0.0295 (6)	0.0177 (6)	0.0082 (5)	0.0200 (6)
O20	0.0418 (7)	0.0324 (7)	0.0269 (6)	0.0004 (6)	0.0070 (5)	0.0086 (5)
O30	0.0575 (10)	0.0587 (10)	0.0500 (9)	0.0222 (8)	−0.0007 (7)	0.0155 (8)
O40	0.0342 (7)	0.0497 (8)	0.0323 (6)	0.0037 (6)	0.0048 (5)	0.0235 (6)
O41	0.0367 (7)	0.0579 (9)	0.0432 (8)	−0.0025 (7)	−0.0056 (6)	0.0230 (7)

### Geometric parameters (Å, º)

**Table:**

N1—C2	1.471 (2)	C26—H26	0.9500
N1—C10	1.478 (2)	C30—C31	1.497 (3)
N1—C20	1.4828 (19)	C30—H30A	0.9900
C2—C3	1.528 (2)	C30—H30B	0.9900
C2—H2A	0.9900	C31—C32	1.392 (3)
C2—H2B	0.9900	C31—C36	1.398 (3)
C3—N4	1.471 (2)	C32—O30	1.370 (3)
C3—H3A	0.9900	C32—C33	1.390 (3)
C3—H3B	0.9900	C33—C34	1.372 (4)
N4—C40	1.476 (2)	C33—H33	0.9500
N4—C30	1.483 (2)	C34—C35	1.361 (4)
C10—C11	1.500 (2)	C34—H34	0.9500
C10—H10A	0.9900	C35—C36	1.392 (3)
C10—H10B	0.9900	C35—H35	0.9500
C11—C16	1.395 (2)	C36—H36	0.9500
C11—C12	1.397 (2)	C40—C41	1.507 (2)
C12—O10	1.361 (2)	C40—H40A	0.9900
C12—C13	1.389 (2)	C40—H40B	0.9900
C13—C14	1.387 (3)	C41—C46	1.384 (2)
C13—H13	0.9500	C41—C42	1.400 (2)
C14—C15	1.383 (3)	C42—O40	1.3556 (18)
C14—H14	0.9500	C42—C43	1.407 (2)
C15—C16	1.389 (2)	C43—C44	1.397 (2)
C15—H15	0.9500	C43—C47	1.456 (2)
C16—H16	0.9500	C44—C45	1.380 (2)
C20—C21	1.509 (2)	C44—H44	0.9500
C20—H20A	0.9900	C45—C46	1.400 (2)
C20—H20B	0.9900	C45—C48	1.505 (2)
C21—C26	1.393 (2)	C46—H46	0.9500
C21—C22	1.401 (2)	C47—O41	1.222 (2)
C22—O20	1.369 (2)	C47—H47	0.9500
C22—C23	1.385 (2)	C48—H48A	0.9800
C23—C24	1.382 (3)	C48—H48B	0.9800
C23—H23	0.9500	C48—H48C	0.9800
C24—C25	1.377 (3)	O10—H10	0.9269
C24—H24	0.9500	O20—H20	0.9386
C25—C26	1.390 (3)	O30—H30	0.9332
C25—H25	0.9500	O40—H40	0.9349
			
C2—N1—C10	111.44 (12)	C25—C26—H26	119.3
C2—N1—C20	112.03 (12)	C21—C26—H26	119.3
C10—N1—C20	111.35 (12)	N4—C30—C31	111.34 (14)
N1—C2—C3	110.69 (12)	N4—C30—H30A	109.4
N1—C2—H2A	109.5	C31—C30—H30A	109.4
C3—C2—H2A	109.5	N4—C30—H30B	109.4
N1—C2—H2B	109.5	C31—C30—H30B	109.4
C3—C2—H2B	109.5	H30A—C30—H30B	108.0
H2A—C2—H2B	108.1	C32—C31—C36	118.15 (19)
N4—C3—C2	116.14 (12)	C32—C31—C30	120.33 (18)
N4—C3—H3A	108.3	C36—C31—C30	121.5 (2)
C2—C3—H3A	108.3	O30—C32—C33	119.1 (2)
N4—C3—H3B	108.3	O30—C32—C31	120.03 (18)
C2—C3—H3B	108.3	C33—C32—C31	120.9 (2)
H3A—C3—H3B	107.4	C34—C33—C32	119.3 (3)
C3—N4—C40	114.03 (13)	C34—C33—H33	120.3
C3—N4—C30	112.54 (12)	C32—C33—H33	120.3
C40—N4—C30	109.74 (13)	C35—C34—C33	121.2 (2)
N1—C10—C11	113.43 (13)	C35—C34—H34	119.4
N1—C10—H10A	108.9	C33—C34—H34	119.4
C11—C10—H10A	108.9	C34—C35—C36	119.9 (2)
N1—C10—H10B	108.9	C34—C35—H35	120.0
C11—C10—H10B	108.9	C36—C35—H35	120.0
H10A—C10—H10B	107.7	C35—C36—C31	120.4 (2)
C16—C11—C12	117.97 (15)	C35—C36—H36	119.8
C16—C11—C10	121.94 (15)	C31—C36—H36	119.8
C12—C11—C10	119.96 (14)	N4—C40—C41	113.41 (13)
O10—C12—C13	122.45 (15)	N4—C40—H40A	108.9
O10—C12—C11	116.65 (15)	C41—C40—H40A	108.9
C13—C12—C11	120.90 (15)	N4—C40—H40B	108.9
C14—C13—C12	119.98 (16)	C41—C40—H40B	108.9
C14—C13—H13	120.0	H40A—C40—H40B	107.7
C12—C13—H13	120.0	C46—C41—C42	118.20 (14)
C15—C14—C13	120.14 (17)	C46—C41—C40	120.76 (14)
C15—C14—H14	119.9	C42—C41—C40	120.98 (14)
C13—C14—H14	119.9	O40—C42—C41	119.10 (14)
C14—C15—C16	119.55 (16)	O40—C42—C43	121.17 (14)
C14—C15—H15	120.2	C41—C42—C43	119.73 (14)
C16—C15—H15	120.2	C44—C43—C42	119.95 (15)
C15—C16—C11	121.45 (16)	C44—C43—C47	119.64 (15)
C15—C16—H16	119.3	C42—C43—C47	120.34 (15)
C11—C16—H16	119.3	C45—C44—C43	121.23 (15)
N1—C20—C21	111.67 (12)	C45—C44—H44	119.4
N1—C20—H20A	109.3	C43—C44—H44	119.4
C21—C20—H20A	109.3	C44—C45—C46	117.50 (15)
N1—C20—H20B	109.3	C44—C45—C48	121.42 (16)
C21—C20—H20B	109.3	C46—C45—C48	121.06 (16)
H20A—C20—H20B	107.9	C41—C46—C45	123.31 (15)
C26—C21—C22	117.90 (16)	C41—C46—H46	118.3
C26—C21—C20	121.25 (15)	C45—C46—H46	118.3
C22—C21—C20	120.77 (14)	O41—C47—C43	124.37 (17)
O20—C22—C23	119.00 (16)	O41—C47—H47	117.8
O20—C22—C21	120.26 (15)	C43—C47—H47	117.8
C23—C22—C21	120.74 (17)	C45—C48—H48A	109.5
C24—C23—C22	120.06 (18)	C45—C48—H48B	109.5
C24—C23—H23	120.0	H48A—C48—H48B	109.5
C22—C23—H23	120.0	C45—C48—H48C	109.5
C25—C24—C23	120.42 (18)	H48A—C48—H48C	109.5
C25—C24—H24	119.8	H48B—C48—H48C	109.5
C23—C24—H24	119.8	C12—O10—H10	112.6
C24—C25—C26	119.46 (18)	C22—O20—H20	109.6
C24—C25—H25	120.3	C32—O30—H30	103.6
C26—C25—H25	120.3	C42—O40—H40	104.9
C25—C26—C21	121.40 (18)		
			
C10—N1—C2—C3	81.42 (15)	C40—N4—C30—C31	72.17 (19)
C20—N1—C2—C3	−153.07 (13)	N4—C30—C31—C32	54.0 (2)
N1—C2—C3—N4	174.78 (13)	N4—C30—C31—C36	−128.35 (18)
C2—C3—N4—C40	72.09 (17)	C36—C31—C32—O30	176.19 (18)
C2—C3—N4—C30	−53.74 (18)	C30—C31—C32—O30	−6.1 (3)
C2—N1—C10—C11	−159.44 (12)	C36—C31—C32—C33	−3.7 (3)
C20—N1—C10—C11	74.67 (16)	C30—C31—C32—C33	174.00 (18)
N1—C10—C11—C16	−117.78 (16)	O30—C32—C33—C34	−178.0 (2)
N1—C10—C11—C12	66.33 (19)	C31—C32—C33—C34	1.9 (3)
C16—C11—C12—O10	−178.35 (15)	C32—C33—C34—C35	1.5 (3)
C10—C11—C12—O10	−2.3 (2)	C33—C34—C35—C36	−2.9 (3)
C16—C11—C12—C13	1.6 (2)	C34—C35—C36—C31	1.0 (3)
C10—C11—C12—C13	177.67 (15)	C32—C31—C36—C35	2.3 (3)
O10—C12—C13—C14	178.56 (17)	C30—C31—C36—C35	−175.38 (17)
C11—C12—C13—C14	−1.4 (3)	C3—N4—C40—C41	67.55 (17)
C12—C13—C14—C15	0.3 (3)	C30—N4—C40—C41	−165.16 (14)
C13—C14—C15—C16	0.5 (3)	N4—C40—C41—C46	54.7 (2)
C14—C15—C16—C11	−0.3 (3)	N4—C40—C41—C42	−128.20 (16)
C12—C11—C16—C15	−0.8 (2)	C46—C41—C42—O40	−177.66 (14)
C10—C11—C16—C15	−176.73 (15)	C40—C41—C42—O40	5.2 (2)
C2—N1—C20—C21	72.32 (16)	C46—C41—C42—C43	1.9 (2)
C10—N1—C20—C21	−162.11 (13)	C40—C41—C42—C43	−175.28 (15)
N1—C20—C21—C26	−146.22 (15)	O40—C42—C43—C44	177.11 (15)
N1—C20—C21—C22	37.1 (2)	C41—C42—C43—C44	−2.4 (2)
C26—C21—C22—O20	179.39 (15)	O40—C42—C43—C47	0.2 (3)
C20—C21—C22—O20	−3.8 (2)	C41—C42—C43—C47	−179.35 (16)
C26—C21—C22—C23	−0.9 (2)	C42—C43—C44—C45	0.3 (3)
C20—C21—C22—C23	175.91 (16)	C47—C43—C44—C45	177.31 (17)
O20—C22—C23—C24	179.02 (16)	C43—C44—C45—C46	2.1 (2)
C21—C22—C23—C24	−0.7 (3)	C43—C44—C45—C48	−176.43 (16)
C22—C23—C24—C25	1.4 (3)	C42—C41—C46—C45	0.7 (2)
C23—C24—C25—C26	−0.4 (3)	C40—C41—C46—C45	177.87 (15)
C24—C25—C26—C21	−1.2 (3)	C44—C45—C46—C41	−2.7 (3)
C22—C21—C26—C25	1.8 (2)	C48—C45—C46—C41	175.86 (16)
C20—C21—C26—C25	−174.92 (16)	C44—C43—C47—O41	−177.20 (19)
C3—N4—C30—C31	−159.71 (15)	C42—C43—C47—O41	−0.3 (3)

### Hydrogen-bond geometry (Å, º)

**Table:**

*D*—H···*A*	*D*—H	H···*A*	*D*···*A*	*D*—H···*A*
O10—H10···O20^i^	0.93	1.80	2.7230 (16)	177
O20—H20···N1	0.94	1.75	2.5928 (17)	148
O20—H20···O10	0.94	2.43	3.0362 (19)	122
O30—H30···N4	0.93	1.94	2.784 (2)	149
O40—H40···O41	0.93	1.76	2.6146 (19)	151

Symmetry code: (i) −*x*+1, −*y*, −*z*.
